# Complete genome sequence of *Odoribacter splanchnicus* type strain (1651/6^T^)

**DOI:** 10.4056/sigs.1714269

**Published:** 2011-04-29

**Authors:** Markus Göker, Sabine Gronow, Ahmet Zeytun, Matt Nolan, Susan Lucas, Alla Lapidus, Nancy Hammon, Shweta Deshpande, Jan-Fang Cheng, Sam Pitluck, Konstantinos Liolios, Ioanna Pagani, Natalia Ivanova, Konstantinos Mavromatis, Galina Ovchinikova, Amrita Pati, Roxane Tapia, Cliff Han, Lynne Goodwin, Amy Chen, Krishna Palaniappan, Miriam Land, Loren Hauser, Cynthia D. Jeffries, Evelyne-Marie Brambilla, Manfred Rohde, John C. Detter, Tanja Woyke, James Bristow, Victor Markowitz, Philip Hugenholtz, Jonathan A. Eisen, Nikos C. Kyrpides, Hans-Peter Klenk

**Affiliations:** 1 DSMZ - German Collection of Microorganisms and Cell Cultures GmbH, Braunschweig, Germany; 2 DOE Joint Genome Institute, Walnut Creek, California, USA; 3 Los Alamos National Laboratory, Bioscience Division, Los Alamos, New Mexico, USA; 4 Biological Data Management and Technology Center, Lawrence Berkeley National Laboratory, Berkeley, California, USA; 5 Oak Ridge National Laboratory, Oak Ridge, Tennessee, USA; 6 HZI – Helmholtz Centre for Infection Research, Braunschweig, Germany; 7 Australian Centre for Ecogenomics, School of Chemistry and Molecular Biosciences, The University of Queensland, Brisbane, Australia; 8 University of California Davis Genome Center, Davis, California, USA

**Keywords:** strictly anaerobic, non-motile, Gram-negative, opportunistic pathogen, mesophilic, chemoorganotrophic, *Porphyromonadaceae*, GEBA

## Abstract

*Odoribacter splanchnicus* (Werner *et al*. 1975) Hardham *et al.* 2008 is the type species of the genus *Odoribacter*, which belongs to the family *Porphyromonadaceae* in the order ‘*Bacteroidales*’. The species is of interest because members of the *Odoribacter* form an isolated cluster within the *Porphyromonadaceae*. This is the first completed genome sequence of a member of the genus *Odoribacter* and the fourth sequence from the family *Porphyromonadaceae*. The 4,392,288 bp long genome with its 3,672 protein-coding and 74 RNA genes and is a part of the *** G****enomic* *** E****ncyclopedia of* *** B****acteria and* *** A****rchaea * project.

## Introduction

Strain 1651/6^T^ (= DSM 20712 = ATCC 29572 = JCM 15291) is the type strain of *Odoribacter splanchnicus* [1,2]. Currently, there are three species placed in the genus *Odoribacter* [1]. The generic name derives from the Latin noun *odor* meaning *smell* and the Neo-Latin word *bacter* meaning *a rod*, referring to a rod of (bad) smell [2]. The species epithet is derived from the Greek plural noun *splanchna* meaning *innards*, referring to the internal organs as the site of isolation [2]. *O. splanchnicus* strain 1651/6^T^ was isolated as *Bacteroides splanchnicus* from a human, abdominal abscess by Werner and Reichertz in 1971 [3] and described in 1975 [4]. The species was first validly published as *B. splanchnicus* due to a number of shared characteristics with the members of the genus *Bacteroides*. However, the organism differs from other *Bacteroides* species in a number of important biochemical characteristics [5] and shows less than 20% relatedness in the homology of 16S rRNA genes compared to the *B. fragilis* group [6]. In 1994, through further studies of the phylogenetic structure of the bacteroides subgroup it became clear that *B. splanchnicus* did not belong to the genera *Bacteroides*, *Prevotella* or *Porphyromonas*, but fell just outside these three major clusters [7]. Finally, in 2008, the new genus *Odoribacter* was described and *B. splanchnicus* was reclassified as its new type species [2]. Additional isolates of *O. splanchnicus* have been obtained from stool specimens and surgically removed appendices [2]; in one case of pelviperitonitis the organism was isolated from a blood sample and peritoneal pus [8]. In general, *O. splanchnicus* can be described as an inhabitant of the human intestine that has the potential to become an opportunistic pathogen. Here we present a summary classification and a set of features for *O. splanchnicus* 1651/6^T^, together with the description of the complete genomic sequencing and annotation.

## Classification and features

A representative genomic 16S rRNA sequence of strain 1651/6^T^ was compared using NCBI BLAST under default settings (*e.g*., considering only the high-scoring segment pairs (HSPs) from the best 250 hits) with the most recent release of the Greengenes database [9] and the relative frequencies of taxa and keywords (reduced to their stem [10]) were determined, weighted by BLAST scores. The most frequently occurring genera were *Bacteroides* (43.5%), *Odoribacter* (37.9%), *Alistipes* (15.2%) and *Brumimicrobium* (3.4%) (21 hits in total). Regarding the two hits to sequences from members of the species, the average identity within HSPs was 99.7%, whereas the average coverage by HSPs was 97.9%. Regarding the two hits to sequences from other members of the genus, the average identity within HSPs was 93.4%, whereas the average coverage by HSPs was 42.5%. The highest-scoring environmental sequence was EF401000 ('human fecal clone SJTU D 04 48'), which showed an identity of 99.8% and an HSP coverage of 98.2%. The most frequently occurring keywords within the labels of environmental samples which yielded hits were 'human' (13.4%), 'biopsi' (7.4%), 'mucos' (7.1%), 'fecal' (6.1%) and 'colon' (5.3%) (229 hits in total). The most frequently occurring keyword within the labels of environmental samples which yielded hits of a higher score than the highest scoring species was 'fecal/human' (50.0%) (27 hits in total).

**Figure 1:**
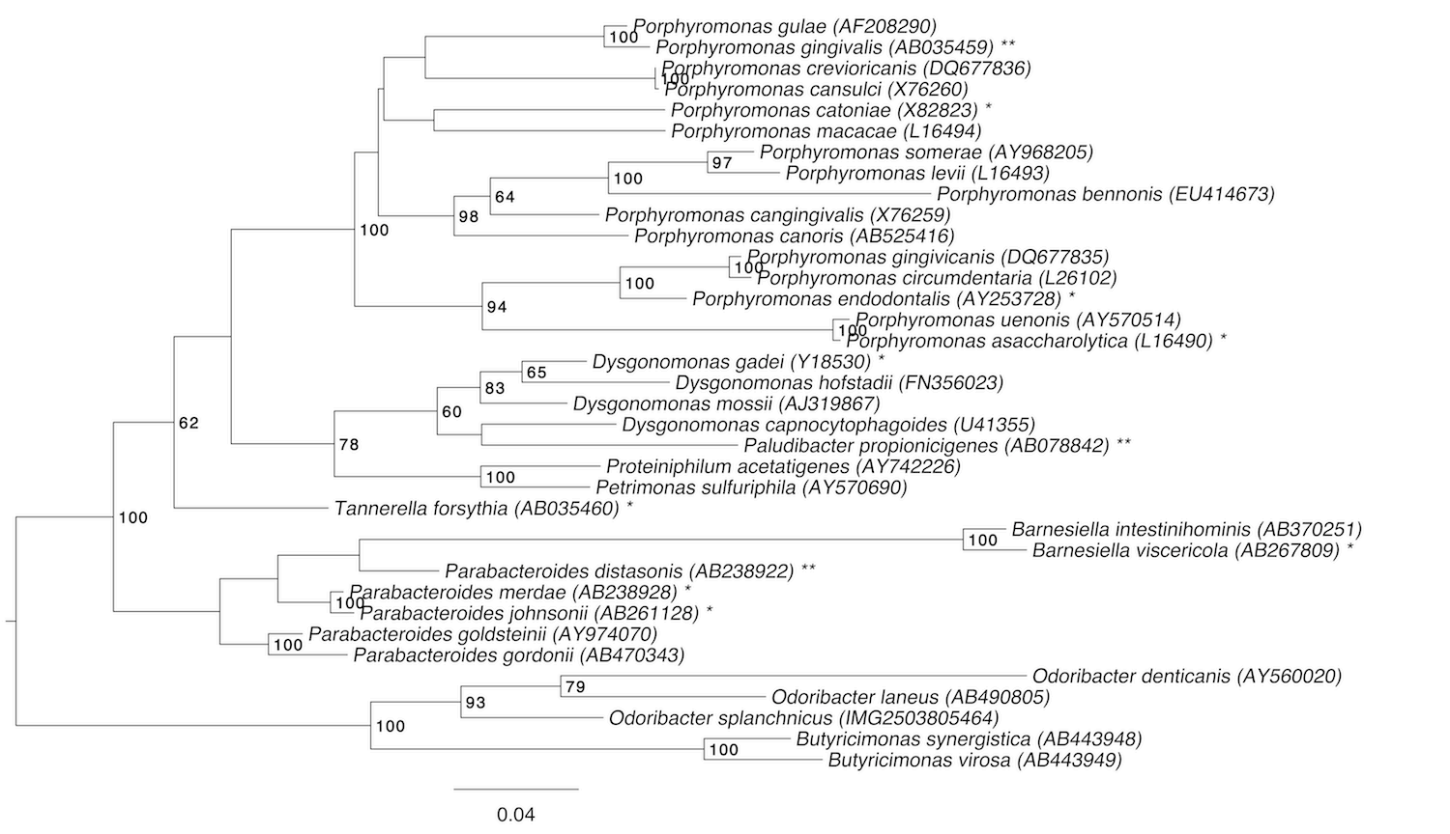
Phylogenetic tree highlighting the position of *O. splanchnicus* relative to the other type strains within the family *Porphyromonadaceae*. The tree was inferred from 1,401 aligned characters [11,12] of the 16S rRNA gene sequence under the maximum likelihood criterion [13]. Rooting was done initially using the midpoint method [14] and then checked for its agreement with the current classification (Table 1). The branches are scaled in terms of the expected number of substitutions per site. Numbers to the right of bifurcations are support values from 250 bootstrap replicates [15] if larger than 60%. Lineages with type strain genome sequencing projects registered in GOLD [16] are labeled by one asterisk, published genomes by two asterisks [17-19].

Figure 1 shows the phylogenetic neighborhood of *O. splanchnicus* in a 16S rRNA based tree. The sequences of the four 16S rRNA gene copies in the genome differ from each other by up to eight nucleotides, and differ by up to nine nucleotides from the previously published 16S rRNA sequence (L16496), which contains nine ambiguous base calls

**Figure 2:**
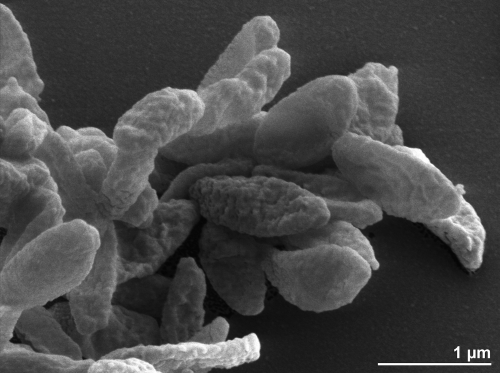
Scanning electron micrograph of *O. splanchnicus* 1651/6^T^

The cells of *O. splanchnicus* generally have the shape of short rods (0.7 × 1.0-5.0 µm) which occur singly or in lightly associated groups (Figure 2). They can also be pleomorphic. *O. splanchnicus* is a Gram-negative, non-pigmented and non spore-forming bacterium (Table 1). The organism is described as non-motile and only ten genes associated with motility have been found in the genome (see below). *O. splanchnicus* grows well at 37°C, is strictly anaerobic, chemoorganotrophic and is able to ferment glucose, fructose, galactose, arabinose, lactose and mannose but does not utilize sucrose, rhamnose, trehalose or salicin [4,5]. The organism does not reduce nitrate but it produces indole from tryptophan and hydrolyzes esculin [28]. *O. splanchnicus* does not require hemin for growth but is highly stimulated by its presence and does not show hemolysis on blood agar. Growth is enhanced by the addition of 20% bile. Major fermentation products are acetic acid, propionic acid and succinic acid; butyric acid, isovaleric acid and isobutyric acid are produced in small amounts [4,29]. When amino acids are used as carbon sources, only lysine enables butyrate production [4]. It is known that *O. splanchnicus* possesses highly active pentose phosphate pathway enzymes such as glucose-6-phosphate dehydrogenase and 6-phosphogluconate dehydrogenase as well as active malate dehydrogenase and glutamate dehydrogenase [30]. The organism produces large amounts of hydrogen and H_2_S. Strain 1651/6^T^ is phosphatase, α- and β-galactosidase, α-fucosidase, *N*-acetylglucosaminidase and glutamic acid decarboxylase active and urease and catalase inactive [2]. The organism produces arginine arylamidase, leucyl glycine arylamidase, leucine arylamidase, alanine arylamidase (own, unpublished data) and glycylprolyl arylamidase [31]. *O. splanchnicus* is reported to grow in the presence of aminoglycosides and polymyxins (minimum inhibitory concentration (MIC) value greater than 60 µg/ml); chloramphenicol, penicillins and cephalosporins show bacteriostatic activity (5-40 µg/ml). The organism is susceptible to tetracyclines, lincomycin, clindamycin, rifampicin and erythromycin (MIC values less than 0.5 µg/ml) [4,28].

**Table 1:** Classification and general features of *O. splanchnicus* 1651/6^T^ according to the MIGS recommendations [20].

MIGS ID	Property	Term	Evidence code
	Current classification	Domain *Bacteria*	TAS [21]
		Phylum '*Bacteroidetes*'	TAS [22]
		Class '*Bacteroidia*'	TAS [23,24]
		Order '*Bacteroidales*'	TAS [25]
		Family '*Porphyromonadaceae*'	TAS [25]
		Genus *Odoribacter*	TAS [2]
		Species *Odoribacter splanchnicus*	TAS [2]
		Type strain 1651/6	TAS [4]
	Gram stain	negative	TAS [4]
	Cell shape	rod-shaped	TAS [4]
	Motility	non-motile	TAS [4]
	Sporulation	none	TAS [4]
	Temperature range	mesophile	TAS [4]
	Optimum temperature	37°C	TAS [4]
	Salinity	normal	TAS [4]
MIGS-22	Oxygen requirement	strictly anaerobic	TAS [4]
	Carbon source	carbohydrates; nitrogenous substrates	TAS [4]
	Energy metabolism	chemoorganotroph	TAS [4]
MIGS-6	Habitat	*Homo sapiens*, gastrointestinal tract	TAS [4]
MIGS-15	Biotic relationship	free-living	NAS
MIGS-14	Pathogenicity	opportunistic pathogen	TAS [4]
	Biosafety level	2	TAS [26]
	Isolation	abdominal abscess	TAS [3]
MIGS-4	Geographic location	Germany	TAS [3]
MIGS-5	Sample collection time	1971 or before	TAS [3]
MIGS-4.1	Latitude	not reported	NAS
MIGS-4.2	Longitude	not reported	NAS
MIGS-4.3	Depth	not reported	NAS
MIGS-4.4	Altitude	not reported	NAS

Evidence codes - IDA: Inferred from Direct Assay (first time in publication); TAS: Traceable Author Statement (i.e., a direct report exists in the literature); NAS: Non-traceable Author Statement (i.e., not directly observed for the living, isolated sample, but based on a generally accepted property for the species, or anecdotal evidence). These evidence codes are from of the Gene Ontology project [27]. If the evidence code is IDA, then the property was directly observed by one of the authors or an expert mentioned in the acknowledgements.

### Chemotaxonomy

Little chemotaxonomic information is available for strain 1651/6^T^. It possesses *meso*-diaminopimelic acid in its peptidoglycan [30], sphingophospholipids as polar lipids [32] and the sole menaquinone present is MK-9 [30]. The major fatty acids found are *iso-*C_15:0_, C_14:0_, *anteiso-*C_15:0_ and C_16:03-OH_ [30].

## Genome sequencing and annotation

### Genome project history

This organism was selected for sequencing on the basis of its phylogenetic position [33], and is part of the *** G****enomic* *** E****ncyclopedia of* *** B****acteria and* *** A****rchaea * project [34]. The genome project is deposited in the Genomes On Line Database [16] and the complete genome sequence is deposited in GenBank. Sequencing, finishing and annotation were performed by the DOE Joint Genome Institute (JGI). A summary of the project information is shown in Table 2.

**Table 2:** Genome sequencing project information

**MIGS ID**	**Property**	**Term**
MIGS-31	Finishing quality	Finished
MIGS-28	Libraries used	Three genomic libraries: one 454 pyrosequence standard library, one 454 PE library (8 kb insert size), one Illumina library
MIGS-29	Sequencing platforms	Illumina GAii, 454 GS FLX Titanium
MIGS-31.2	Sequencing coverage	521.0 × Illumina; 31.5 × pyrosequence
MIGS-30	Assemblers	Newbler version 2.3-PreRelease-10-21-2009, Velvet version 0.7.63, phrap version 4.24
MIGS-32	Gene calling method	Prodigal 1.4, GenePRIMP
	INSDC ID	CP002544
	Genbank Date of Release	February 28, 2011
	GOLD ID	Gc01667
	NCBI project ID	43469
	Database: IMG-GEBA	2503754021
MIGS-13	Source material identifier	DSM 20712
	Project relevance	Tree of Life, GEBA

### Growth conditions and DNA isolation

*O. splanchnicus* 1651/6^T^, DSM 20712, was grown anaerobically in DSMZ medium 110 (Chopped meat medium with carbohydrates) [35] at 37°C. DNA was isolated from 0.5-1 g of cell paste using Jetflex Genomic DNA Purification kit (GENOMED 600100) following the standard protocol as recommended by the manufacturer, but adding 20 µL proteinase K for 45 min lysis at 58ºC. DNA is available through the DNA Bank Network [36].

### Genome sequencing and assembly

The genome was sequenced using a combination of Illumina and 454 sequencing platforms. All general aspects of library construction and sequencing can be found at the JGI website [37]. Pyrosequencing reads were assembled using the Newbler assembler version 2.3-PreRelease-10-21-2009 (Roche). The initial Newbler assembly consisting of 57 contigs in eight scaffolds was converted into a phrap [38] assembly by making fake reads from the consensus, to collect the read pairs in the 454 paired end library. Illumina GAii sequencing data (2,241.8 Mb) was assembled with Velvet, version 0.7.63 [39] and the consensus sequences were shredded into 1.5 kb overlapped fake reads and assembled together with the 454 data. The 454 draft assembly was based on 138 Mb 454 draft data and all of the 454 paired end data. Newbler parameters are -consed -a 50 -l 350 -g -m -ml 20. The Phred/Phrap/Consed software package [38] was used for sequence assembly and quality assessment in the subsequent finishing process. After the shotgun stage, reads were assembled with parallel phrap (High Performance Software, LLC). Possible mis-assemblies were corrected with gapResolution [37], Dupfinisher, or sequencing cloned bridging PCR fragments with subcloning or transposon bombing (Epicentre Biotechnologies, Madison, WI) [40]. Gaps between contigs were closed by editing in Consed, by PCR and by Bubble PCR primer walks (J.-F.Chang, unpublished). A total of 65 additional reactions were necessary to close gaps and to raise the quality of the finished sequence. Illumina reads were also used to correct potential base errors and increase consensus quality using a software Polisher developed at JGI [41]. The error rate of the completed genome sequence is less than 1 in 100,000. Together, the combination of the Illumina and 454 sequencing platforms provided 552.5 × coverage of the genome. The final assembly contained 389,415 pyrosequence and 33,128,505 Illumina reads.

### Genome annotation

Genes were identified using Prodigal [42] as part of the Oak Ridge National Laboratory genome annotation pipeline, followed by a round of manual curation using the JGI GenePRIMP pipeline [43]. The predicted CDSs were translated and used to search the National Center for Biotechnology Information (NCBI) nonredundant database, UniProt, TIGR-Fam, Pfam, PRIAM, KEGG, COG, and InterPro databases. Additional gene prediction analysis and functional annotation was performed within the Integrated Microbial Genomes - Expert Review (IMG-ER) platform [44].

## Genome properties

**Figure 3:**
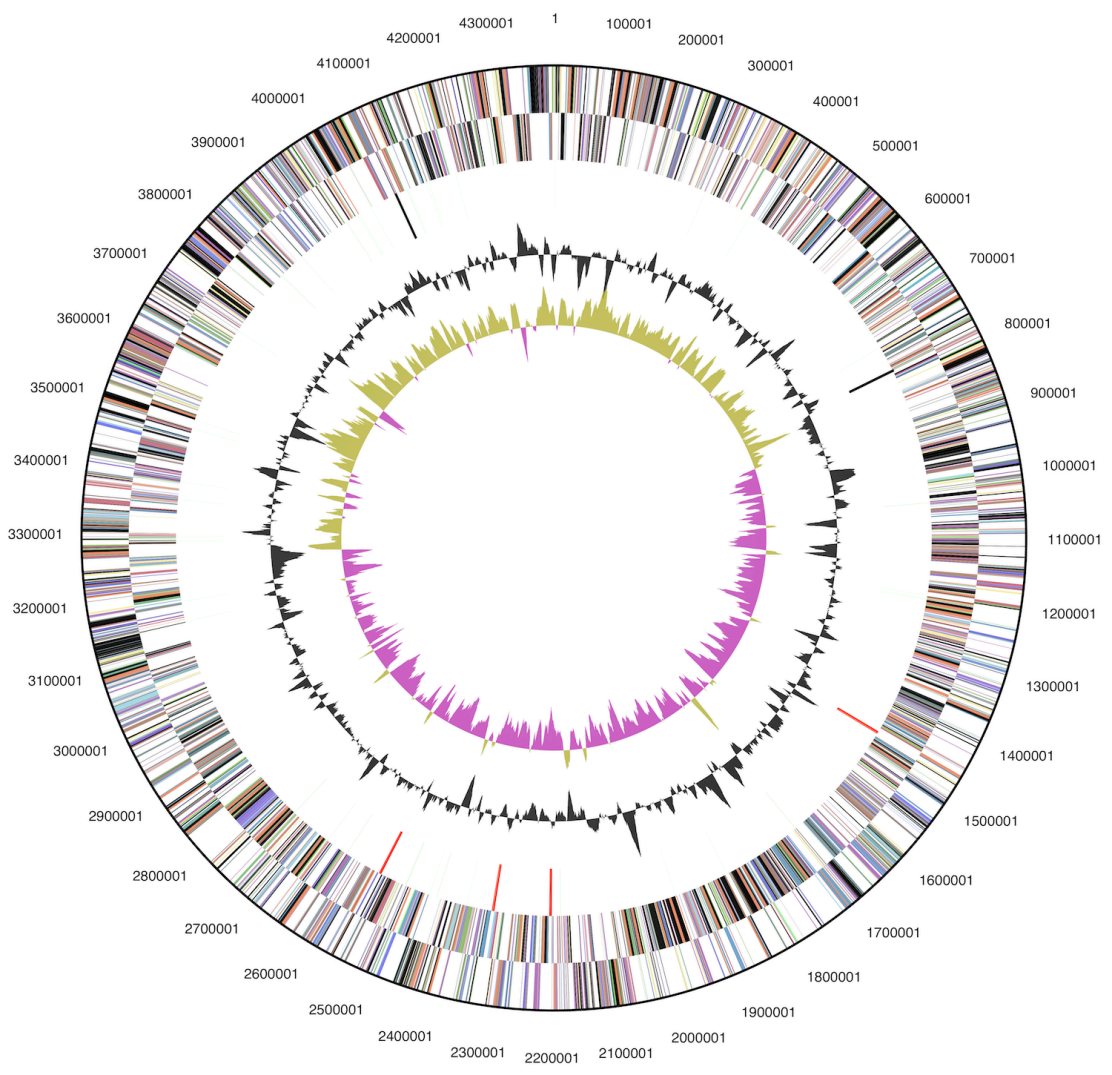
Graphical circular map of the chromosome. From outside to the center: Genes on forward strand (color by COG categories), Genes on reverse strand (color by COG categories), RNA genes (tRNAs green, rRNAs red, other RNAs black), GC content, GC skew.

The genome consists of a 4,392,288 bp long chromosome with a G+C content of 43.4% (Table 3 and Figure 3). Of the 3,746 genes predicted, 3,672 were protein-coding genes, and 74 RNAs; 175 pseudogenes were also identified. The majority of the protein-coding genes (61.2%) were assigned with a putative function while the remaining ones were annotated as hypothetical proteins. The distribution of genes into COGs functional categories is presented in Table 4.

**Table 3:** Genome Statistics

**Attribute**	Value	% of Total
Genome size (bp)	4,392,288	100.00%
DNA coding region (bp)	3,824,553	87.07%
DNA G+C content (bp)	1,904,432	43.36%
Number of replicons	1	
Extrachromosomal elements	0	
Total genes	3,746	100.00%
RNA genes	74	1.98%
rRNA operons	4	
Protein-coding genes	3,672	98.02%
Pseudo genes	175	4.67%
Genes with function prediction	2,291	61.16%
Genes in paralog clusters	734	19.59%
Genes assigned to COGs	2,252	60.12%
Genes assigned Pfam domains	2,523	67.35%
Genes with signal peptides	909	24.27%
Genes with transmembrane helices	823	21.97%
CRISPR repeats	1	

**Table 4:** Number of genes associated with the general COG functional categories

Code	value	%age	Description
J	149	5.9	Translation, ribosomal structure and biogenesis
A	0	0.0	RNA processing and modification
K	188	7.5	Transcription
L	161	6.4	Replication, recombination and repair
B	0	0.0	Chromatin structure and dynamics
D	23	0.9	Cell cycle control, cell division, chromosome partitioning
Y	0	0.0	Nuclear structure
V	67	2.7	Defense mechanisms
T	144	5.7	Signal transduction mechanisms
M	215	8.6	Cell wall/membrane/envelope biogenesis
N	10	0.4	Cell motility
Z	0	0.0	Cytoskeleton
W	0	0.0	Extracellular structures
U	48	2.1	Intracellular trafficking, secretion, and vesicular transport
O	134	5.3	Posttranslational modification, protein turnover, chaperones
C	164	6.5	Energy production and conversion
G	111	4.4	Carbohydrate transport and metabolism
E	175	7.0	Amino acid transport and metabolism
F	62	2.5	Nucleotide transport and metabolism
H	126	5.2	Coenzyme transport and metabolism
I	62	2.5	Lipid transport and metabolism
P	216	8.6	Inorganic ion transport and metabolism
Q	24	1.0	Secondary metabolites biosynthesis, transport and catabolism
R	280	11.2	General function prediction only
S	149	5.9	Function unknown
-	1,494	39.9	Not in COGs
